# Progress in Medicine

**Published:** 1895-03-30

**Authors:** 


### Progress in Medicine.

### DISEASES OF THE CIRCULATORY SYSTEM.

(*Continued from page 443.*)

**Endocarditis** has been traced of late to a number of septic causes, and found to arise in the course of various diseases, where little or no relation was formerly suspected. Gonorrhœa is one[1] of those affections which may give rise to either a simple or malignant endocarditis. This has only been noted as yet in some thirty cases, mostly those associated with joint lesions. Erysipelas, again, may produce[2] either the simple or malignant form of the disease, the latter due to actual settlements of the streptococcus on the valves, the former to some unknown action, possibly the result of a toxin. Myocarditis and two types of pericarditis occasionally appear in the course of an attack of this disease. Chorea has long been known to be accompanied at times by endocarditis. Osler now states that "there is no known disease in which endocarditis is so constantly found post-mortem as chorea. It is exceptional to find the heart healthy." Again, "there is no other disease, not even acute rheumatism, which is so frequently accompanied with valvulitis";[3] and "in a considerable proportion of cases, much larger indeed than has hitherto been supposed, the endocarditis lays the foundation of organic heart disease," though independent of, and not associated with, acute arthritis. Pneumo ɔia: Bignami[4] reports on five cases originating in the diplococcus of this disease, and refers to previous cases of Weichselbaum and others, especially as to the frequency of lesions from this cause in the right ventricle. Netter[5] records an instance, with bacterial examination of the blood, caused by infection through a wound in the leg, and not through the air passages. Acute Rheumatism: Leyden, after referring to previous researches, mentions that[6] he has found in five cases of rheumatic endocarditis, not of the malignant variety, a small diplococcus, which he cultivated on ascitic fluid, and which may be the cause of articular rheumatism. The malignant forms seem to be due to a mixed infection. Tuberculosis: Girandeau reviews a number of cases occurring in cachetic patients,[7] such as hemiplegics and those suffering from cancer, especially of ulcerating types, where secondary infections exist. He notes the frequency with which phlegmasia dolens and phlebitis have been precursors of the endocarditis, and discusses Teissier's conclusion that vegetative endocarditis in tubercular patients is due to secondary infection, while the granular form, especially found in acute phthisis, is produced by tubercular bacilli. Mouisset, Londe and Petit record other cases.[8] Girandeau shows reason for believing that even the vegetative form may be tubercular, while Leyden and Fraenkel have found the bacillus in some cases of the ulcerative type.

**Pericarditis.**—Josserand[9] points to an early sign to be found before friction sounds can be heard. The pulmonary second sound is more intense than that on the right side, also louder and clanging. The difference of the shock can often be felt by the hand. The great importance of adherent pericardium in children is well worked out by Theodore Fisher. It is far more common than valvular disease, and not only causes great enlargement of the heart, but is the most fatal and serious form of heart disease before the age of fifteen. The ordinary diagnostic signs are of little value, but he points out that there is often a presystolic or diastolic bruit not produced by stenosis, which can often be heard in these cases[10]. The murmurs are, he thinks, always low pitched, and accompany a systolic murmur of a long duration, while the first heart sound is not sharp and short.[11] If the murmur is diastolic it may be heard over the right ventricle or may form part of a *bruit de galop*.

**Nerve Disorders.**—Dubois[12] reports a case of paroxysmal tachycardia relieved by occasional pressure on the vagus, the pulse falling from 140 to 80, and remaining so for some days. Five cases where extreme slowness of the pulse seemed to cause epileptiform fits are discussed by Bristowe,[13] who raises the question what is the greatest pause between the beats which can take place without convulsions from cerebral anæmia. In one case he noted an interval of seven seconds without attacks. Hanot[14] gives a similar case with atheromatous lesions in the cerebellum, which he thinks were the principal cause. Other cases are reported by Calthrop and Fisher.

The heart in gout presents few physical signs,[15] but there may be severe attacks of dyspnœa, pain, palpitation, and faintness, out of proportion to the signs, which may indicate at most a large feeble organ. The symptoms, too, come on most frequently after some sudden exertion, and the prognosis is favourable. Mitchell Bruce lays stress on the importance of a spare diet, baths, purgatives, gentle exercise, and cardiac tonics, since high tension is not habitual, and the heart is weak and neurotic, rather than hypertrophied and vigorous. The absurd views of the French Academy as to the danger of cycling were contradicted at the Medical Society where B. W. Richardson[16] spoke from long practical experience and careful observations. He considers that in moderation it only benefits the healthy heart; it is useful in certain cases where the action is feeble and some fatty degeneration exists, just as Schott's methods of treatment strengthen the weak heart. The pulse rate is remarkably quickened during the exercise, and the work got out of the heart is phenomenally great. Over-exertion has more evil effect on worn-out arteries than on the heart, but varicose veins seem to be benefited by moderate cycling. Overstrain and alcoholic stimulants should be avoided. On the whole, the effects on the heart seem to be distinctly favourable.

[1] Central f. In, Med., 14, 1894. [2] Ditto, 16, 1894 [3] Osler on Chorea. [4] Pract., Aug. [5] Med. Week, June 1. [6] Med. Week, July 13. [7] Med. Week, Oct. 19. [8] B.M.J., July 14. [9] B.M.J., Dec. 8. [10] Bristol M.C.J., June. [11] Med. Week, Aug. 10. [12] Am. J. Med. Sc., Nov. [13] Lancet, Sept. 22. [14] Med. Week, June 22. [15] Pract. Jan., 1895. [16] Lancet, Jan. 19, 1895.

